# Health system responsiveness to older adults with functional limitations and socio-economic vulnerability in India

**DOI:** 10.1080/16549716.2026.2678648

**Published:** 2026-06-26

**Authors:** Thi Vinh Nguyen, Marie Ishida, Ajay Mahal, Sumit Kane

**Affiliations:** Nossal Institute for Global Health, Melbourne School of Population and Global Health, The University of Melbourne, Carlton, Australia

**Keywords:** Functional limitations, health system responsiveness, India, older adults, socio-economic vulnerability

## Abstract

**Background:**

Health system responsiveness (HSR) is a core health system goal yet remains underexamined for older adults with functional limitations and socio-economic vulnerability in India.

**Objective:**

To present a novel assessment of HSR among older adults aged 60+ with functional limitations and socio-economic vulnerability in India across six domains: prompt attention, dignity, communication, confidentiality, choice of providers, and quality of amenities.

**Methods:**

We analysed data from the nationally representative Longitudinal Ageing Study in India Wave 1 (2017–2018), including 16,659 older adults for outpatient care and 2,358 for inpatient care. Multivariable linear regressions assessed the association between HSR and functional limitations, multimorbidity, and socio-economic factors. Functional limitations were defined as one or more limitations in Activities of Daily Living and/or Instrumental Activities of Daily Living. Overall HSR score was calculated by summing across domains.

**Results:**

Median HSR score was 24 (range 6–30), equivalent to approximately 75 on a 0–100 scale, for outpatient and inpatient care. Older adults with functional limitations experienced poorer responsiveness in outpatient (β = −0.62; 95%CI: −0.84, −0.41) and inpatient care (β = −0.62; 95%CI: −1.14, −0.09). Negative associations were observed in public facilities, while in private facilities this was significant only for outpatient care. Among those with functional limitations, lower castes and lower economic status reported poorer responsiveness.

**Conclusions:**

Older adults with functional limitations in India experience poorer HSR, with worse care among lower castes and poorer groups. These findings suggest the presence of ableism within the Indian health system that needs to be systematically identified and tackled to ensure equitable and responsive healthcare.

## Paper Context

- **Main findings:** Older adults with functional limitations in India experience poorer health system responsiveness in both outpatient and inpatient care, with disparities particularly pronounced in public facilities and further amplified by socio-economic disadvantage.
- **Added knowledge:** This study provides one of the first nationally representative assessments across India of health system responsiveness among older adults with functional limitations, highlighting how ageing, functional limitations, and socio-economic vulnerability intersect to shape non-clinical care experiences.
- **Global health impact for policy and action:** Strengthening health system responsiveness through policy action and health system reforms to address structural ableism is essential to provide equitable, disability-responsive care for India’s ageing population, especially those with functional limitations and socio-economic disadvantage.

## Background

Currently, older adults, that is, those aged 60 and above, make up approximately 14% of the world’s population; this is expected to increase to 22% by 2050 [1,2]. India is currently home to approximately 150 million older adults, and this number is expected to rise to more than 200 million in the next 10 years and to more than 250 million by 2050 [2]. As India ages, its health system will need to change to address ageing-related health challenges, particularly chronic conditions, comorbidities, and functional limitations [3–5]. In response, several national initiatives, such as the National Programme for Health Care of the Elderly, the National Policy for Senior Citizens, and recently expanded coverage for older adults under Ayushman Bharat, have sought to strengthen healthcare access and financial protection for older populations [6,7]. However, beyond access and coverage, ensuring that care is delivered in a responsive and equitable manner remains a critical challenge. From a health systems perspective, these challenges are likely to be two-fold. One, to provide good-quality medical services for chronic conditions, comorbidities, and functional limitations/disabilities to older adults in an equitable and affordable manner. Second, to provide these services in a ‘responsive’ manner.

The World Health Organization (WHO) outlines eight domains of health systems responsiveness (HSR): prompt attention, dignity, autonomy, confidentiality, communication, choice of care providers, access to social networks and support during care, and quality of basic amenities [8,9]. A responsive health system enables timely and effective use of healthcare services, which, in turn, contributes to improved health outcomes and health equity [8,10,11]. We argue that a good test of a health system’s state of responsiveness is its performance vis-à-vis those with greater healthcare needs and those socio-economically disadvantaged. We define healthcare need and vulnerability along two axes. First, in the context of older adults, we operationalise those with greater healthcare need as those with one or more limitations in Activities of Daily Living (ADL) (dressing, walking, bathing, eating, getting in or out of bed, using toilet) and/or in Instrumental Activities of Daily Living (IADL) (cooking, shopping, making telephone calls, taking medicines, doing work around the house or garden, managing money, getting around); and the presence of one or more chronic illnesses. Second, in the context of India, we define socio-economic vulnerability as being from a lower economic status or hailing from the lowest social castes, i.e. those from the Scheduled Castes or the Scheduled Tribes. Such an analysis we contend fills a critical gap in the literature by focusing on the intersection of ageing, socio-economic vulnerability, and health systems responsiveness – an area that has received limited empirical attention in the Indian context [12].

Evidence from low- and middle-income countries (LMICs) indicates that responsiveness is generally lower in public healthcare facilities compared to private ones [4,13]. Several socio-demographic factors have been found to influence HSR, such as sex, residence, and wealth [4,14,15]. For example, a study in South Africa identified that males and urban residents reported higher levels of responsiveness in inpatient care [13]. Similar patterns were observed across five countries (China, Ghana, India, Russia, South Africa) in the WHO Study on global AGEing and Adult Health (SAGE) Wave 1 (2007–2010), although these differences were not statistically significant after adjusting for covariates [3]. Higher income is also consistently associated with better responsiveness [3,14]. There is some evidence of age-related patterning too, with older people reporting better responsiveness. For instance, a study from Tanzania found that being of older age was associated with a lower likelihood of poor responsiveness [14]; similarly, in South Africa, older people aged 80 years and above experienced higher levels of responsiveness compared to younger older people [13].

While much of the literature focuses on socio-demographic determinants of responsiveness, few studies have examined if having functional limitations and chronic diseases affects how older people experience their care encounters. Evidence from the SAGE study suggests no significant differences in responsiveness between individuals with acute versus chronic conditions [3], whereas other analyses indicate that older adults with disabilities experience significantly lower responsiveness than those without [12].

In this paper, using data from the Longitudinal Ageing Study in India (LASI) [16] we answer the following question: ‘Is the Indian health system responsive to older adults with functional limitations, multimorbidity, and socio-economic vulnerability?’ Our aim is to generate insights into if and how the Indian health system addresses the needs of vulnerable older adults and to identify areas for targeted improvements in equitable and responsive care.

## Methods

### Data source

We analysed data from Wave 1 of the Longitudinal Ageing Study India (LASI), conducted during 2017–2018 across all states and union territories of India [16]. LASI is a publicly accessible, nationally representative longitudinal household survey that collects information on health, healthcare use, and social and economic wellbeing of older adults [17,18]. LASI employs a multistage stratified area probability cluster sampling design, comprising four stages in urban areas and three stages in rural areas to ensure representativeness at the national and state levels [18].

### Study sample

Respondents in the LASI survey are adults aged 45 and above. We focused on older individuals aged 60 years and above who reported receiving either outpatient care or inpatient care at least once in the past 12 months. Inpatient care refers to at least one overnight stay in a health facility, while outpatient care refers to visits to a healthcare provider that did not involve an overnight stay, including home-based care provided by healthcare workers. The analysis was restricted to respondents who answered all questions related to responsiveness for either outpatient or inpatient care.

**Figure 1.**
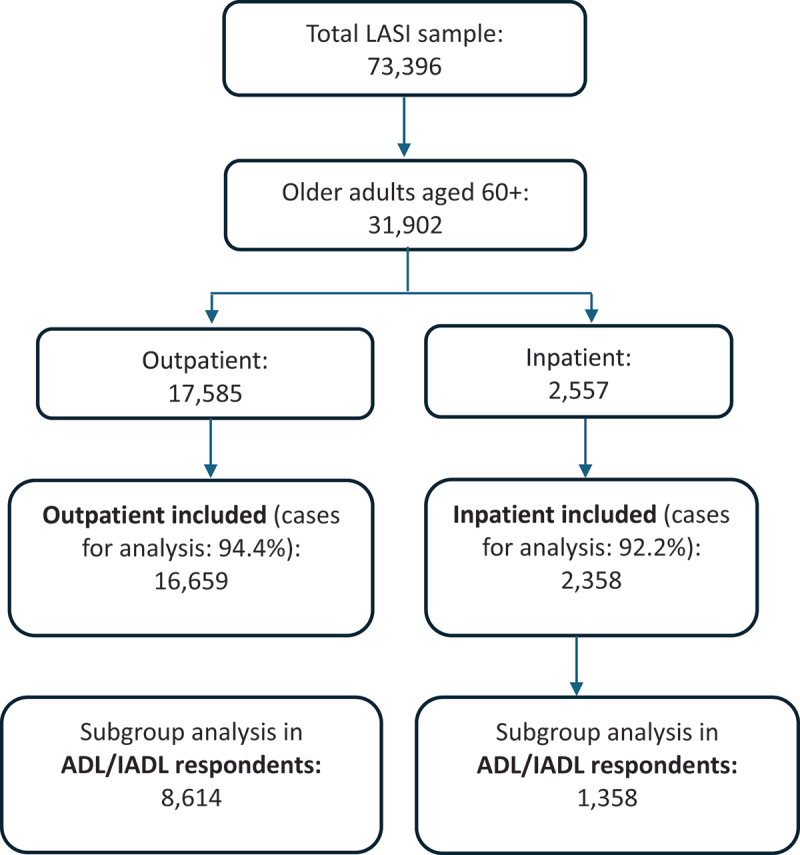
Study sample size. **Note**: LASI: Longitudinal Aging Study in India. ADL: Activities of Daily Living. IADL: Instrumental Activities of Daily Living.

Figure 1 presents the sampling flowchart. The full LASI dataset includes 73,396 individuals, representing all 36 states and union territories of India. We conducted a complete-case analysis. Observations with missing data on outcome variables or relevant covariates were excluded from the analysis. As our study focused on analysing social caste, respondents who selected ‘no caste/tribe’ and ‘don’t know’ (98 for inpatient care and 534 for outpatient care) were also excluded from the analysis. After these exclusions, the final analysed sample comprised 16,659 respondents (94.4%) for outpatient care, and 2,358 respondents (92.2%) for inpatient care.

For the subgroup analysis focusing on individuals with functional limitations, we identified respondents with at least one difficulty in Activities of Daily Living (ADL) or Instrumental Activities of Daily Living (IADL). Among these, 8,614 respondents were included in the outpatient care analysis, and 1,358 respondents in the inpatient care analysis.

## Measures

### Health systems responsiveness

The outcome variable was health systems responsiveness. Six domains of HSR were collected in LASI: (i) prompt attention, (ii) dignity, (iii) communication, (iv) confidentiality, (v) choice of providers, and (vi) quality of basic amenities, separately for outpatient and inpatient care [19]. Information on two domains, autonomy and access to support networks, was not collected. In LASI, each domain was assessed separately based on the respondents’ most recent visit to an inpatient or outpatient service within the past 12 months. Accordingly, analyses of HSR were conducted separately for inpatient and outpatient services. Respondents were asked to rate their experience in each domain using the following questions:

- Prompt attention: ‘Rate your experience about the length of time you waited before being attended to’
- Dignity: ‘Rate your experience of being treated respectfully’
- Communication: ‘Rate your experience of how clearly health care providers explained things to you’
- Confidentiality: ‘Rate your experience the way the health care staff ensured that you could talk privately to providers’
- Choice of providers: ‘Rate your experience of seeing a health care provider of your choice’
- Quality of basic amenities: ‘Rate your experience of cleanliness in the health facility’

Responses were recorded on a five-point Likert scale (1 = very good, 2 = good, 3 = moderate, 4 = bad, and 5 = very bad). For ease of interpretation, the scale was reverse coded so that higher scores indicated better responsiveness (5 = very good to 1 = very bad).

Overall HSR score was calculated by summing scores across the six domains, resulting in a total score ranging from 6 to 30, with higher scores indicating better perceived responsiveness across domains. Each domain was given equal weight in the construction of the index; this is consistent with empirical studies of health system responsiveness [13,14]. Overall HSR score was treated as continuous rather than dichotomised, as dichotomisation can reduce statistical power and alter the true relationships between correlated predictors [20,21]. The HSR scale demonstrated high internal consistency, with an average Cronbach’s alpha of 0.899 for outpatient care and 0.909 for inpatient care, indicating good reliability.

### Health conditions

Self-reported limitations in ability, i.e. Activities of Daily Living (ADL), Instrumental Activities of Daily Living (IADL), and the presence of chronic disease were used to measure the health condition of respondents. Respondents who reported difficulty in conducting any ADL and/or IADL were coded as 1 and coded as 0 otherwise. The Longitudinal Ageing Study India (LASI) assessed chronic conditions based on respondents’ self-reported diagnoses to the question: ‘Has any health professional ever diagnosed you with the following chronic conditions or diseases?’ The conditions included hypertension, diabetes, cancer, chronic lung diseases, chronic heart diseases, stroke, arthritis or rheumatism, neurological or psychiatric problems, and high cholesterol. The presence of chronic conditions was classified into three: 1 if respondents reported one chronic condition, 2 if respondents reported two or more chronic conditions, and 0 otherwise.

### Covariates

Several socio-economic and demographic factors were included as covariates in the analysis. Age was treated as a continuous variable. Categorical variables included sex (male, female), residence (urban, rural), years of schooling (none, less than 5 years complete, 6–9 years complete, 10+ years complete), marital status (currently married, currently not married), religion (Hindu, Muslim, others). Caste was classified into three categories: Upper castes (including those who belong to other backward classes, and those not belonging to Scheduled Castes or Scheduled Tribes), Scheduled Caste, and Scheduled Tribe. Economic status (poorest, poorer, middle, richer, richest) was measured using household quintiles based on monthly per capita consumption expenditure (MPCE), which is widely considered a more reliable measure of economic status than income, which tends to be underreported in LMIC settings [18].

### Statistical analysis

We first described the characteristics of respondents seeking outpatient and inpatient care, including demographic, socio-economic, and health conditions. Health system responsiveness across the six domains was then examined according to functional limitations (ADL/IADL difficulty) and chronic disease status.

Bivariable regression was used to estimate unadjusted associations between the overall HSR score and each covariate (Table 2: model 0a for outpatient care and model 0b for inpatient care). Multivariable models were used to examine associations between overall HSR score and functional limitations, chronic disease status, and socio-demographic covariates. Covariates included in the models were residence, age, sex, education, marital status, religion, caste, economic status, and health insurance (Table 2: model 1a for outpatient care and model 1b for inpatient care). Covariates included in the multivariable models were selected based on theoretical considerations and prior literature on HSR [22]. To account for geographic heterogeneity in health systems and socioeconomic conditions across India, multivariable models additionally adjusted for states/territories of residence (Table 2: model 2a for outpatient care and model 2b for inpatient care).

Multicollinearity was assessed using variance inflation factors, with all values below 2, indicating no evidence of collinearity. The linearity assumption for continuous predictors was assessed and found to be reasonable. Robust standard errors were used to account for non-normality and heteroskedasticity; in large samples, minor deviations from normality generally do not substantially affect inference [23]. Results are presented as β coefficients with 95% confidence intervals (95% CI) and *p*-values, separately for outpatient and inpatient care. To facilitate the interpretation of effect sizes, we conducted supplementary analyses using a standardised version of the HSR score, with mean = 0 and standard deviation = 1.

Subgroup analyses (Table 3: models 3a, 3b for outpatient care and models 3c, 3d for inpatient care) were conducted among respondents with functional limitations to examine whether associations with socio-economic vulnerability differed within this group. Additional stratified analyses (Table S4 supplementary file: models 4a, 4b for outpatient care and models 4c, 4d for inpatient care) were performed by facility type (public vs. private) to explore potential differences. All analyses applied LASI sampling weights and were conducted using Stata 18.

## Results

### Characteristics of respondents

Among respondents aged 60+ who used outpatient and inpatient care in the past 12 months, the majority lived in rural areas, were female, had no schooling, were currently married, Hindu, and from upper castes. Functional limitations and chronic conditions were more common among inpatient users (61.5% and 72%, respectively) than outpatient users (54.4% and 59.5%, respectively). Detailed sample characteristics are provided in Table 1.

**Table 1.** Socio-demographic characteristics of respondents.

Characteristics	Outpatient (n = 16,659)		Inpatient (n = 2,358)	
	Frequency	% (weighted)	Frequency	% (weighted)
**Categorical variables**				
**Residence**				
Rural	10,889	71.14	1,532	68.74
Urban	5,770	28.86	826	31.26
**Sex**				
Female	8,910	53.42	1,136	50.44
Male	7,749	46.58	1,222	49.56
**Age group (year)**				
60–69	10,077	58.56	1,338	55.19
70–79	4,948	31.06	766	33.48
80+	1,634	10.38	254	11.33
**Education**				
None	8,726	55.49	1,167	52.95
Less than 5 years complete	2,044	12.09	328	14.2
6–9 years complete	3,294	18.27	491	19.52
10+ years complete	2,595	14.16	372	13.32
**Marital status**				
Currently not married	6,129	38.01	791	35.38
Currently married	10,530	61.99	1,567	64.62
**Religion**				
Hindu	12,706	83.23	1766	80.38
Muslim	2,056	10.32	276	12.47
Others	1,897	6.45	316	7.15
**Caste**				
Upper Castes	11,830	73.96	1,647	73.68
Scheduled Caste	2,978	19.68	375	19.39
Scheduled Tribe	1,851	6.36	336	6.93
**Quintile**				
Poorest	3,042	19.42	311	14.81
Poorer	3,416	21.44	409	18.49
Middle	3,380	21.13	430	18.81
Richer	3,462	20.19	484	20
Richest	3,359	17.83	724	27.88
**Health insurance**				
No	13,114	80.94	1,722	75.86
Yes	3,545	19.06	636	24.14
**ADL/IADL**				
No difficulty	8,045	45.6	1,000	38.53
At least one difficulty	8,614	54.4	1,358	61.47
**Presence of chronic diseases**				
None	6,103	40.46	621	28.02
Single	5,422	32.06	761	32.1
Multiple	5,134	27.48	976	39.87
**Continuous variables**				
**Age**	Median: 67 1IQR: 63 3IQR: 73 Min: 60 Max: 116		Median: 68 1IQR: 63 3IQR: 74 Min: 60 Max: 111	

### Health system responsiveness by functional limitations and chronic diseases

**Figure 2.**
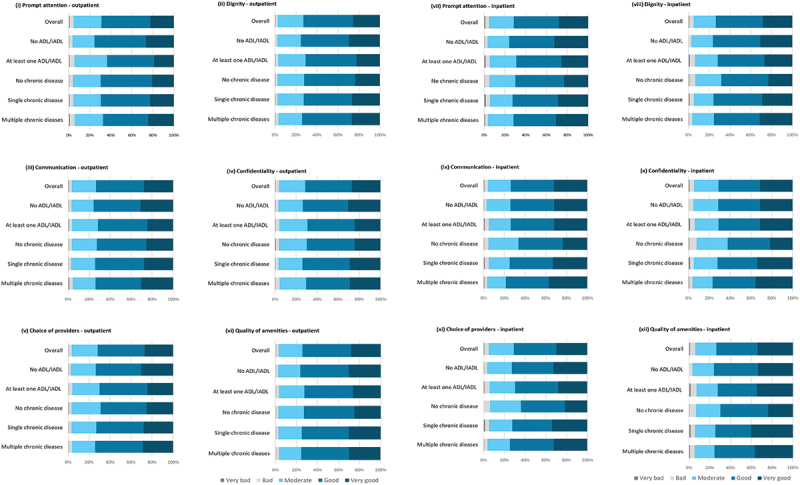
Health system responsiveness by functional limitations and chronic diseases. **Note**: This figure presents descriptive proportions. Confidence intervals are reported in regression table S2, S3 in Supplementary file.

Across all six responsiveness domains, good/very good responsiveness for each domain was reported consistently less among those with functional limitations compared to those without. Good/very good responsiveness ranged from 63.4% for prompt attention of outpatient care to 73.7% for communication of inpatient care among those with functional limitations. Respondents with functional limitations were more likely to report bad/very bad responsiveness compared to those without, consistently for both outpatient and inpatient care. The highest rates of bad/very bad responsiveness were observed for ‘quality of amenities’ (7.32%) and ‘dignity’ (6.17%) during inpatient visits for respondents with functional limitations. Care experience of those with chronic diseases varied: outpatient users with multimorbidity reported poorer responsiveness, whereas the opposite was observed for inpatient users (Figure 2).

### Health system responsiveness and functional limitations

The median overall HSR score was 24 (IQR: 21–26) for outpatient care and 24 (IQR: 21–27) for inpatient care on a scale of 6–30, equivalent to approximately 75 on a 0–100 scale. Table 2 presents the results of bivariable (unadjusted) and multivariable regression analyses examining associations between overall HSR and functional limitations and chronic diseases, adjusted for socio-demographic characteristics, i.e. residence, age, sex, education, marital status, religion, caste, economic status, and health insurance status, for both outpatient and inpatient care (models 1a and 1b). In the adjusted model, respondents with functional limitations reported poorer overall responsiveness for outpatient care (β = −0.61; 95%CI: −0.83, −0.38) and inpatient care (β = −0.54; 95%CI: −1.07, −0.01). After additionally adjusting for state/territory (models 2a and 2b), the magnitude of the associations increased slightly (outpatient: β = −0.62; 95% CI: −0.84, −0.41; inpatient: β = −0.62; 95% CI: −1.14, −0.09).

**Table 2.** Overall health system responsiveness and functional limitations.

Covariates	Outpatient ( n = 16,659)			Inpatient (n = 2358)		
	Unadjusted Coefficient (95% CI) (Model 0a)	Adjusted Coefficient (95% CI) (Model 1a)	Adjusted Coefficient (95% CI) (Model 2a)	Unadjusted Coefficient (95% CI) (Model 0b)	Adjusted Coefficient (95% CI) (Model 1b)	Adjusted Coefficient (95% CI) (Model 2b)
**ADL/IADL**						
No	ref	ref	ref	ref	ref	ref
At least one	-0.78*** (−0.98, −0.56)	-0.61*** (−0.83, −0.38)	-0.62*** (−0.84, −0.41)	−0.52 (−1.09, 0.05)	-0.54* (−1.07, −0.01)	-0.62* (−1.14, −0.09)
**Presence of chronic diseases**						
None	ref	ref	ref	ref	ref	ref
Single	0.32** (0.09, 0.57)	0.17 (−0.05, 0.39)	0.04 (−0.18, 0.26)	1.04** (0.27, 1.82)	0.83* (0.12, 1.53)	0.78* (0.07, 1.49)
Multiple	0.28* (0.01, 0.56)	−0.08 (−0.38, 0.21)	-0.29* (−0.56, −0.01)	1.32*** (0.69, 1.95)	0.94** (0.29, 1.58)	0.79* (0.14, 1.43)
**Residence**						
Rural	ref	ref	ref	ref	ref	ref
Urban	0.84*** (0.56, 1.12)	0.38** (0.10, 0.67)	0.30* (0.05, 0.55)	1.13 *** (0.51, 1.75)	0.63* (0.02, 1.24)	0.24 (−0.40, 0.89)
**Sex**						
Female	ref	ref	ref	ref	ref	ref
Male	0.01 (−0.21, 0.22)	-0.53*** (−0.80, −0.25)	-0.47*** (−0.73, −0.22)	−0.33 (−0.91, 0.24)	−0.45 (−1.02, 0.13)	−0.53 (−1.10, 0.05)
**Age**	−0.00 (−0.01, 0.01)	0.01 (−0.00, 0.03)	0.02 (−0.00, 0.04)	−0.04 (−0.08, 0.01)	−0.03 (−0.07, 0.01)	−0.02 (−0.06, 0.02)
**Education level**						
No schooling	ref	ref	ref	ref	ref	ref
Less than 5 year complete	0.31 (−0.03, 0.66)	0.29 (−0.07, 0.65)	0.20 (−0.15, 0.56)	0.47 (−0.73, 1.66)	0.18 (−0.85, 1.21)	0.08 (−0.92, 1.07)
5–9 years complete	0.7***(0.39, 1.01)	0.50** (0.17, 0.82)	0.56** (0.24, 0.87)	0.35 (−0.37, 1.07)	−0.04 (−0.76, 0.68)	−0.03 (−0.71, 0.64)
10 or more years complete	1.67*** (1.26, 2.07)	1.32*** (0.85, 1.79)	1.37*** (0.98, 1.76)	1.78*** (1.09, 2.48)	1.17** (0.44, 1.91)	1.29** (0.55, 2.04)
**Marriage**						
Currently not married	ref	ref	ref	ref	ref	ref
Currently married	0.45*** (0.24, 0.67)	0.42** (0.15, 0.69)	0.25* (0.00, 0.50)	0.1 (−0.55–0.74)	−0.07 (−0.72, 0.58)	−0.01 (−0.64, 0.63)
**Religion**						
Hindu	ref	ref	ref	ref	ref	ref
Muslim	−0.11 (−0.40, 0.17)	−0.02 (−0.31, 0.28)	−0.02 (−0.34, 0.29)	0.64 (−0.23, 1.51)	0.37 (−0.52, 1.26)	0.63 (−0.31, 1.56)
Others	0.37* (0.02, 0.73)	0.42* (0.06, 0.79)	-0.56** (−0.97, −0.15)	−0.53 (−1.93, 0.87)	−0.23 (−1.48, 1.02)	-1.67* (−3.08, −0.26)
**Caste**						
Upper Castes	ref	ref	ref	ref	ref	ref
Scheduled Caste	-0.71*** (−0.97, −0.45)	-0.41** (−0.68, −0.14)	-0.32* (−0.57, −0.07)	-1.58*** (−2.3, −0.78)	-1.08** (−1.81, −0.35)	-0.80* (−1.50, −0.10)
Scheduled Tribe	-0.55** (−0.89, −0.2)	−0.24 (−0.59, 0.12)	−0.19 (−0.56, 0.18)	−1.08 (−2.22, 0.07)	−0.76 (−1.84, 0.32)	−0.75 (−1.97, 0.47)
**Economic status**						
Higher	ref	ref	ref	ref	ref	ref
Lower	-0.57*** (−0.79, −0.36)	-0.36** (−0.57, −0.14)	-0.42*** (−0.62, −0.22)	-1. 09 *** (−1.7, −0.48)	-0.83** (−1.41, −0.25)	-0.74** (−1.30, −0.18)
**Health insurance**						
No	ref	ref	ref	ref	ref	ref
Yes	0.2 (−0.01, 0.5)	0.13 (−0.15, 0.42)	0.23 (−0.09, 0.56)	0.21 (−0.43, 0.86)	0.27 (−0.32, 0.86)	0.03 (−0.56, 0.62)
States			Included			Included
**Constant**		22.41*** (20.96, 23.85)	21.54*** (19.99, 23.10)		25.44*** (22.37, 28.50)	22.66*** (18.99, 26.33)

Note: *Significant at *p* < 0.05, **significant at *p* < 0.01, ***significant at *p* < 0.001.

Models 0a,b: Unadjusted estimates are based on bivariable regression models.

Models 1a,b: Adjusted estimates are based on multivariable regression models including all covariates listed in the table (without states/territories).

Models 2a,b: Adjusted estimates are based on multivariable regression models including all covariates listed in the table and states/territories (coefficients not shown).

Consistent with the above findings, supplementary analyses using a standardised HSR score (mean = 0, standard deviation = 1) produced similar results (Supplementary Table S1). In these models, functional limitations were associated with a 0.16 standard deviation decrease in outpatient responsiveness and a 0.14 standard deviation decrease in inpatient responsiveness.

Additional analyses by individual responsiveness domains (Supplementary Table S2 for outpatient care and S3 for inpatient care) showed broadly consistent patterns, although some variation across domains was observed. Significant associations between functional limitations and poorer responsiveness were found across all six domains for outpatient care and across three domains, i.e. prompt attention, dignity, and choice of providers, for inpatient care.

A contrasting pattern was observed in the association between overall HSR and chronic disease status between inpatient care and outpatient care. For inpatient care, compared with respondents without chronic diseases, those with one chronic disease (β = 0.78; 95% CI: 0.07, 1.49) and those with multiple chronic diseases (β = 0.79; 95% CI: 0.14, 1.43) reported better responsiveness. In contrast, for outpatient care, those with multiple chronic diseases reported slightly poorer responsiveness (β = −0.29; 95% CI: −0.56, −0.01).

Regarding socio-economic characteristics, respondents from lower caste groups (Scheduled Caste) reported poorer responsiveness for both outpatient care (β = −0.32; 95% CI: −0.57, −0.07) and inpatient care (β = −0.80; 95% CI: −1.50, −0.10). Similarly, respondents in lower economic status rated responsiveness more poorly for outpatient care (β = −0.42; 95% CI: −0.62, −0.22) and inpatient care (β = −0.74; 95% CI: −1.30, −0.18).

In addition, urban residence, being female, higher educational attainment, being currently married, and being Hindu (religion) were associated with better overall HSR for outpatient care. For inpatient care, higher educational attainment and being Hindu were associated with better overall HSR.

To assess whether the association between overall HSR and functional limitations and chronic diseases differed by facility type, we conducted multivariable regression analyses separately for public and private healthcare facilities as a sensitivity analysis (Table S4 Supplementary File). In public facilities, functional limitations were significantly associated with overall HSR for both outpatient and inpatient care. In contrast, in private facilities, a significant association was observed for outpatient care only. These findings suggest that the association between functional limitations and overall HSR may differ by facility type, with more consistent associations observed in public facilities than in private facilities. With respect to chronic diseases, a significant association with overall HSR was observed only for multimorbidity in public outpatient care; patients with multimorbidity reported poorer overall HSR.

### Responsiveness to individuals with functional limitations, further examined by economic status and caste

We further examined the overall HSR within a subgroup of respondents with functional limitations. Multivariable regression analyses showed that within this subgroup, lower economic status was associated with poorer responsiveness for both outpatient and inpatient care (Table 3). Specifically, respondents with lower economic status reported a lower HSR for outpatient care (β = −0.57, 95%CI: −0.83, −0.31) and inpatient care (β = −1.05, 95%CI: −1.85, −0.26). Regarding Caste, Scheduled Caste respondents reported lower HSR compared with Upper Castes for inpatient care (β = −1.27, 95%CI: −2.25, −0.29). By contrast, among respondents without functional limitations, no significant associations were observed between HSR and caste or economic status. These findings suggest that socio-economic disadvantage is associated with poorer responsiveness of the health system.

**Table 3.** HSR in subgroup of ADL/IADL difficulties for outpatient and inpatient care.

Covariates	Outpatient with functional limitation (n = 8,614)	Inpatient with functional limitation (n = 1,358)	Outpatient without functional limitation (n = 8,045)	Inpatient without functional limitation (n = 1,000)
	Adjusted Coefficient (95% CI) (Model 3a)	Adjusted Coefficient (95% CI) (Model 3b)	Adjusted Coefficient (95% CI) (Model 3c)	Adjusted Coefficient (95% CI) (Model 3d)
**Economic status**				
Higher	ref	ref	ref	ref
Lower	-0.57*** (−0.83, −0.31)	-1.05** (−1.85, −0.26)	−0.19 (−0.47, 0.09)	−0.48 (−1.31, 0.35)
**Caste**				
Upper Castes	ref	ref	ref	ref
Scheduled Caste	−0.34 (−0.70, 0.02)	-1.27* (−2.25, −0.29)	−0.27 (−0.61, 0.07)	−0.24 (−1.25, 0.76)
Scheduled Tribe	−0.13 (−0.57, 0.32)	−1.08 (−2.39, 0.23)	−0.35 (−0.96, 0.26)	−0.77 (−2.53, 0.98)
**Residence**				
Rural	ref	ref	ref	ref
Urban	0.36* (0.02, 0.70)	0.95* (0.05, 1.85)	0.17 (−0.16, 0.51)	−0.40 (−1.23, 0.43)
**Sex**				
Female	ref	ref	ref	ref
Male	−0.33 (−0.68, 0.03)	−0.18 (−0.94, 0.59)	-0.60*** (−0.90, −0.29)	−0.73 (−1.59, 0.12)
**Age**	**0.02 (−0.01, 0.04)**	−0.03 (−0.09, 0.03)	0.02 (−0.00, 0.04)	−0.02 (−0.08, 0.03)
**Education level**				
No schooling	ref	ref	ref	ref
Less than 5 year complete	0.30 (−0.19, 0.80)	−0.23 (−1.58, 1.12)	−0.11 (−0.54, 0.31)	0.44 (−0.75, 1.63)
5–9 years complete	0.55* (0.09, 1.01)	0.23 (−0.80, 1.25)	0.53** (0.14, 0.92)	−0.45 (−1.44, 0.54)
10 or more years complete	0.95* (0.23, 1.68)	1.06 (−0.02, 2.13)	1.52*** (1.11, 1.94)	1.68** (0.59, 2.77)
**Marriage**				
Currently not married	ref	ref	ref	ref
Currently married	0.43* (0.08, 0.79)	−0.45 (−1.29, 0.39)	0.04 (−0.28, 0.35)	0.38 (−0.55, 1.32)
**Religion**				
Hindu	ref	ref	ref	ref
Muslim	−0.26 (−0.67, 0.15)	0.25 (−1.12, 1.63)	0.31 (−0.17, 0.78)	1.11 (−0.28, 2.49)
Others	-0.80* (−1.41, −0.19)	-1.97* (−3.76, −0.17)	−0.30 (−0.86, 0.26)	−0.84 (−2.67, 0.99)
**Health insurance**				
No	ref	ref	ref	ref
Yes	0.19 (−0.29, 0.68)	−0.01 (−0.90, 0.88)	0.21 (−0.14, 0.57)	0.22 (−0.59, 1.02)
**States/Territories**	Included	Included	Included	Included
**Constant**	20.67*** (18.51, 22.83)	22.86*** (18.16, 27.57)	21.93*** (20.16, 23.71)	25.13*** (19.88, 30.37)

**Note**: *Significant at *p* < 0.05, **significant at *p* < 0.01, ***significant at *p* < 0.001. Adjusted estimates are based on multivariable regression models including all covariates listed in the table and states/territories (coefficients not shown).

## Discussion

Using data from the first wave of the nationally representative Longitudinal Ageing Study in India (LASI), we examined the health system responsiveness to older adults aged 60+. By focusing on older adults with greater healthcare needs, specifically those with functional limitations and/or chronic diseases, and on socio-economic vulnerability, including individuals with lower economic status and lower castes, this study contributes to the limited but growing literature on the state of HSR for older adults in LMICs.

A key finding of this study is that respondents with functional limitations consistently reported poorer overall HSR and poorer experiences across multiple domains for both outpatient and inpatient care. Although the effect sizes are small in standardised HSR (−0.14 to −0.16 standard deviations in responsiveness), they are consistent across care settings and reflect systematic disadvantages in responsiveness among older adults with functional limitations. This effect size was comparable to, or larger than, those observed for several socio-economic factors, highlighting that having functional limitation is an important correlate of poorer responsiveness. While few studies have examined the association between HSR and having functional limitations, our findings align with Rahman et al. [12], who analysed the SAGE dataset collected during 2007 and 2010 in six countries, including India. Importantly, our analysis provides a more comprehensive national picture for India, as LASI was conducted across all 36 states and union territories of the country, whereas SAGE India covered only 6 states. Peltzer et al. also examined experiences of care of those with functional limitations in South Africa and found no significant differences. This discrepancy may reflect differences in measurement, as functional limitations were assessed using a single item on difficulty with work or household activities over the past 30 days, as well as contextual differences in how the health system is organised in India and South Africa [13].

These differences notwithstanding, inequities in healthcare access between people with and without disabilities in LMICs [24,25] are well documented. Healthcare providers in LMICs often view people with disabilities as being passive and lacking autonomy and ability to make informed decisions [24,26]. Ageing intersects with functional limitations, further exacerbating disparities for older adults with such limitations. This intersection could help explain why the responsiveness of both outpatient and inpatient care is poorer among older adults with functional limitations, in comparison to those with no functional limitations. This finding potentially points to entrenched ableism within the Indian health system [27], and highlights that health policymakers, managers, and service providers need to pay greater attention to the care being delivered to older adults with functional limitations and disabilities to ensure more equitable and responsive care, and to systematically identify and tackle ableism across the health system [24].

Our analyses further demonstrate that the association between overall HSR and functional limitations and chronic disease status varied by facility type. Functional difficulties were consistently associated with poorer HSR in public facilities for both outpatient and inpatient care, whereas in private facilities this association was observed only for outpatient care. These patterns suggest that facility type modifies how functional limitations translate into patient experiences. The association observed in public facilities may reflect limited capacity to address non-clinical needs related to functional limitations, such as mobility support and tailored communication. In contrast, the absence of a significant association in private inpatient care may indicate, expectedly, as Lakin & Kane have argued, better institutional capacities and greater financial incentives to serve clients, including those with functional limitations, during hospitalisation [28].

That respondents with multimorbidity reported better responsiveness for inpatient care may reflect the nature of inpatient services, which typically involve more intensive, coordinated, and continuous interactions with healthcare providers during a defined episode of care. Such settings may offer more structured care processes, closer monitoring, and greater provider attention, which can enhance perceived responsiveness [29,30]. In contrast, this pattern was not observed for outpatient care. Respondents with multimorbidity reported slightly poorer responsiveness, particularly for public facilities. This finding is consistent with studies which have reported that patients with multimorbidity tend to rate responsiveness more poorly [31], but contrasts with evidence showing no association between having chronic diseases and level of responsiveness [3]. These mixed findings emphasise the need for further research to better clarify the direction of the relationship between chronic diseases, particularly multimorbidity and responsiveness in the context that multimorbidity is increasingly common among older adults in LMICs’ rapidly ageing population [32]. These findings also highlight the need for health systems to strengthen responsiveness for patients with chronic conditions in public facilities by improving care coordination and continuity, person-centred care, and support for complex needs, while ensuring that the advantages observed in private inpatient care are equitably extended across all facility types.

The finding that older adults from lower economic status and lower caste groups reported poorer responsiveness reaffirms what the literature from India has consistently highlighted – that the Indian health system has still a long way to go to be equitably responsive to those who are socio-economic vulnerable, i.e. those who are poor, and those who hail from socially disadvantaged backgrounds [33–36]. This pattern is consistent with international evidence, including a six-country study which found that the outpatient health system responsiveness increases with economic status [12]. However, self-reported disparities may in fact be underestimated, as expectations of care are disproportionately shaped by prior experiences, social positions, and cultural norms [37]. Research suggests that people from disadvantaged backgrounds may give higher ratings to lower-quality care due to lower expectations [38,39] or may be reluctant to criticise health providers for fear of being further mistreated or losing out on care. This aligns with the scholarship from India which shows how service expectations are not only shaped by the service’s characteristics but also by broader societal, cultural, and historical contexts [40–42], including caste.

Our analysis also revealed that urban residents and individuals with higher educational attainment reported better HSR compared with those living in rural areas or with no formal schooling. This pattern is consistent with previous evidence from LMICs which shows that urban populations often have greater access to health facilities, shorter travel times, and more opportunities to choose providers, all of which can translate into better experiences of responsiveness [13,43]. Similarly, higher education may empower patients to better navigate the health system and communicate effectively with providers, resulting in more favourable responsiveness ratings [44]. These findings underscore the importance of addressing geographic and educational disparities when designing interventions to improve HSR, particularly for older adults from rural or socio-economically disadvantaged communities. Importantly, our new analysis makes an important contribution to the understanding of how multiple vulnerabilities amongst older individuals, i.e. functional limitations, chronic diseases, economic status, and social caste, intersect to shape how they experience their care encounters with the health system in India. The findings also imply that as the Indian population ages, prioritising the health system’s responsiveness to older adults, especially those with functional limitations and disabilities, multimorbidity, and those from disadvantaged socio-economic backgrounds, is crucial. Achieving this requires health system-level reforms in healthcare infrastructure, resource allocation, and service delivery to enhance access to and responsiveness of care for all older adults in India [45–47].

Our findings should be interpreted in light of ongoing policy efforts in India to strengthen healthcare for older adults, including the National Programme for Health Care of the Elderly and expanded financial protection under Ayushman Bharat [6,7]. While these initiatives have focused on improving access to and affordability of care, our results suggest that inequities in health system responsiveness persist, particularly among older adults with functional limitations and those from socio-economically disadvantaged groups. This indicates that expanding service coverage alone may be insufficient to ensure equitable healthcare experiences. Instead, our findings highlight important gaps in the delivery of care, particularly in domains such as dignity, communication, prompt attention, and choice of providers, which are central to patient-centred care.

Addressing these gaps will require moving beyond access-focused reforms towards embedding responsiveness within health system design and delivery. This could include strengthening provider training on respectful and inclusive care, integrating disability- and age-sensitive communication practices, and implementing routine monitoring of patient experience across diverse population groups. Furthermore, greater policy attention is needed to ensure that existing programmes effectively reach and respond to the needs of the vulnerable older adults, particularly those facing intersecting disadvantages related to disability, poverty, and social caste.

## Limitations

Several limitations should be considered. Firstly, as a cross-sectional study, LASI cannot establish a causal relationship between health system responsiveness and the associated covariates. Second, given that the participants were asked to rate their experiences with healthcare services from the past 12 months, there is some risk of recall bias. Third, the measurement of HSR is based on a subset of domains and survey items available in LASI, which represent a shortened version of the HSR framework also used in large-scale surveys such as the WHO Study on global AGEing and Adult Health (SAGE). While these items capture key aspects of responsiveness, some domains are proxied using relatively narrow indicators (for example, perceived cleanliness as a proxy for the quality of basic amenities), which may not fully reflect the broader conceptual definitions. As such, the HSR index should be interpreted as an approximation of the underlying construct rather than a comprehensive measure. Relatedly, the use of aggregated domain scores assumes that the selected items adequately represent each dimension of responsiveness, although variation in how respondents interpret and report their experiences may influence the estimates. Additionally, the LASI study only assessed six domains of responsiveness; future research should consider additional domains, such as autonomy and access to support networks, to provide a more comprehensive understanding of older adults’ healthcare experience. Fourth, the measurement of chronic conditions and multimorbidity is based on respondents’ self-reported diagnoses to the question of whether a health professional has ever diagnosed them with specific conditions. This approach may be subject to underdiagnosis, particularly among socio-economically disadvantaged groups with more limited access to healthcare. This could lead to an underestimation of health needs in these populations and may bias the estimated associations between socio-economic vulnerability, health status, and health system responsiveness. Lastly, our findings are based on the Indian context and are therefore not directly generalisable to all LMICs, many of which have distinct demographic, social, and health-system characteristics. Nevertheless, the aspects we examine, i.e. functional limitations, socio-economic vulnerability, and health system responsiveness, are conceptually relevant across LMIC settings.

## Conclusions

Our findings reveal that older adults with functional limitations experience significantly poorer responsiveness across outpatient and inpatient care, with particularly pronounced disadvantages among those from lower castes and those who are poor. These patterns highlight persistent inequities in patient experience that extend beyond access to care and reflect deeper structural barriers within the health system. We conclude that tackling entrenched ableism and casteism is essential for equitable and responsive healthcare for older people in India. As India continues to strengthen care for its rapidly ageing population through initiatives such as the National Programme for Health Care of the Elderly, embedding responsiveness within health system performance assessments should be a central priority. Further research is required to explore the complex interactions between ageing, disability, social, and economic disadvantage, and healthcare experience to inform health system improvements in India but also in other ageing societies.

## Supplementary Material

260430_Supplementary_file_clean.docx

## Data Availability

LASI data is publicly available at https://www.iipsindia.ac.in/content/LASI-data
