# Supplementary material for: Health system responsiveness to older adults with functional limitations and socio-economic vulnerability in India
Source: Glob Health Action. 2026 Jun 26;19(1):2678648. doi: 10.1080/16549716.2026.2678648 (PMC13312836; doi:10.1080/16549716.2026.2678648)
Supplement: 260430_Supplementary_file_clean.docx [file ZGHA_A_2678648_SM7088.docx]

**Supplementary file**

**Table S1. Associations between covariates and standardized HSR scores (mean = 0, SD = 1)**

| **Covariates** | **Outpatient (N=16,659)** | **Inpatient (N=2358)** |
| --- | --- | --- |
|  | **Adjusted Coefficient (95% CI)**  **(Model 5a)** | **Adjusted Coefficient (95% CI)**  **(Model 5b)** |
| **ADL/IADL** |  |  |
| No | ref | ref |
| At least one | **-0.16*** (-0.21, -0.10)** | **-0.14* (-0.26, -0.02)** |
| **Presence of chronic diseases** |  |  |
| None | ref | ref |
| Single | 0.01 (-0.04, 0.06) | **0.18* (0.02, 0.35)** |
| Multiple | **-0.07* (-0.14, -0.00)** | **0.18* (0.03, 0.33)** |
| **Residence** |  |  |
| Rural | ref | ref |
| Urban | **0.08* (0.01, 0.14)** | 0.06 (-0.09, 0.21) |
| **Gender** |  |  |
| Female | ref | ref |
| Male | **-0.12*** (-0.18, -0.06)** | -0.12 (-0.25, 0.01) |
| **Age** | 0 (-0.00, 0.01) | -0.00 (-0.01, 0.00) |
| **Education level** |  |  |
| No schooling | ref | ref |
| Less than 5 year complete | 0.05 (-0.04, 0.14) | 0.02 (-0.21, 0.25) |
| 5-9 years complete | **0.14** (0.06, 0.22)** | -0.01 (-0.16, 0.15) |
| 10 or more years complete | **0.35*** (0.25, 0.44)** | **0.30** (0.13, 0.47)** |
| **Marriage** |  |  |
| Currently not married | ref | ref |
| Currently married | **0.06* (0.00, 0.13)** | -0.00 (-0.15, 0.15) |
| **Religion** |  |  |
| Hindu | ref | ref |
| Muslim | -0.01 (-0.08, 0.07) | 0.15 (-0.07, 0.36) |
| Others | **-0.14** (-0.24, -0.04)** | **-0.39* (-0.72, -0.06)** |
| **Caste** |  |  |
| Upper Castes | ref | ref |
| Schedule Caste | **-0.08* (-0.14, -0.02)** | **-0.19* (-0.35, -0.02)** |
| Schedule Tribe | -0.05 (-0.14, 0.05) | -0.17 (-0.46, 0.11) |
| **Economic status** |  |  |
| Higher | ref | ref |
| Lower | **-0.11*** (-0.15, -0.06)** | **-0.17** (-0.30, -0.04)** |
| **Health insurance** |  |  |
| No | ref | ref |
| Yes | 0.06 (-0.02, 0.14) | 0.01 (-0.13, 0.14) |
| **State/territories** | Included | Included |
| **Constant** | **-0.56** (-0.95, -0.17)** | **-0.31 (-1.16, 0.54)** |

**Note:** **Significant at p<0.05, **significant at p<0.01, ***significant at p<0.001.*  *Adjusted estimates are based on multivariable regression models including all covariates listed in the table and states/territories (coefficients not shown). HSR scores were standardized to have mean 0 and standard deviation. Coefficients represent differences in standard deviation units.*

**Table S2: HSR domains and functional limitations for OUTPATIENT care**

| **Covariates** | **Outpatient (N=16,659)** | | | | | |
| --- | --- | --- | --- | --- | --- | --- |
|  | **Prompt attention** | **Dignity** | **Communication** | **Confidentiality** | **Choice of providers** | **Quality of amenities** |
| **ADL/IADL** |  |  |  |  |  |  |
| No | ref | ref | ref | ref | ref | ref |
| At least one | **-0.19*** (-0.23, -0.15)** | **-0.11*** (-0.15, -0.07)** | **-0.08*** (-0.13, -0.04)** | **-0.08** (-0.13, -0.03)** | **-0.09*** (-0.13, -0.04)** | **-0.07** (-0.11, -0.03)** |
| **Presence of chronic diseases** |  |  |  |  |  |  |
| None | ref | ref | ref | ref | ref | ref |
| Single | -0.01 (-0.06, 0.03) | -0.02 (-0.06, 0.03) | -0.01 (-0.06, 0.03) | 0.04 (-0.00, 0.09) | 0.03 (-0.02, 0.07) | 0.01 (-0.03, 0.06) |
| Multiple | -0.05 (-0.11, 0.00) | **-0.06* (-0.11, -0.01)** | -0.06 (-0.13, 0.00) | -0.05 (-0.11, 0.01) | -0.02 (-0.07, 0.04) | -0.05 (-0.10, 0.00) |
| **Residence** |  |  |  |  |  |  |
| Rural | ref | ref | ref | ref | ref | ref |
| Urban | 0.02 (-0.03, 0.07) | 0.03 (-0.02, 0.08) | 0.05 (-0.01, 0.10) | 0.04 (-0.02, 0.09) | **0.08** (0.02, 0.13)** | **0.09*** (0.04, 0.14)** |
| **Gender** |  |  |  |  |  |  |
| Female | ref | ref | ref | ref | ref | ref |
| Male | **-0.09** (-0.14, -0.04)** | **-0.06* (-0.11, -0.02)** | **-0.09** (-0.15, -0.03)** | **-0.07* (-0.13, -0.02)** | **-0.08** (-0.12, -0.03)** | **-0.08** (-0.12, -0.03)** |
| **Age** | **0.00* (0.00, 0.01)** | 0 (-0.00, 0.01) | 0 (-0.00, 0.01) | **0.00* (0.00, 0.01)** | 0 (-0.00, 0.00) | 0.00 (-0.00, 0.01) |
| **Education level** |  |  |  |  |  |  |
| No schooling | ref | ref | ref | ref | ref | ref |
| Less than 5 year complete | 0.02 (-0.05, 0.10) | 0.05 (-0.02, 0.11) | 0.04 (-0.04, 0.11) | 0.03 (-0.04, 0.10) | 0.04 (-0.03, 0.11) | 0.03 (-0.05, 0.10) |
| 5-9 years complete | **0.07* (0.00, 0.13)** | **0.11*** (0.05, 0.17)** | **0.09** (0.02, 0.16)** | **0.10** (0.04, 0.17)** | **0.09* (0.02, 0.15)** | **0.10** (0.04, 0.16)** |
| 10 or more years complete | **0.18*** (0.09, 0.27)** | **0.25*** (0.18, 0.32)** | **0.24*** (0.16, 0.31)** | **0.24*** (0.13, 0.36)** | **0.24*** (0.16, 0.31)** | **0.22*** (0.15, 0.29)** |
| **Marriage** |  |  |  |  |  |  |
| Currently not married | ref | ref | ref | ref | ref | ref |
| Currently married | 0.05 (-0.00, 0.10) | 0.03 (-0.02, 0.08) | 0.06* (0.00, 0.11) | 0.05 (-0.01, 0.10) | 0.03 (-0.02, 0.08) | 0.03 (-0.01, 0.08) |
| **Religion** |  |  |  |  |  |  |
| Hindu | ref | ref | ref | ref | ref | ref |
| Muslim | -0.06 (-0.13, 0.00) | 0.00 (-0.06, 0.06) | 0.01 (-0.05, 0.08) | 0.00 (-0.06, 0.07) | -0.00 (-0.07, 0.07) | 0.02 (-0.04, 0.09) |
| Others | **-0.09* (-0.17, -0.01)** | -0.07 (-0.15, 0.01) | **-0.08* (-0.17, -0.00)** | -0.04 (-0.12, 0.04) | **-0.15** (-0.24, -0.06)** | **-0.13** (-0.22, -0.04)** |
| **Caste** |  |  |  |  |  |  |
| Upper Castes | ref | ref | ref | ref | ref | ref |
| Schedule Caste | **-0.05* (-0.10, -0.00)** | -0.04 (-0.09, 0.01) | **-0.06* (-0.11, -0.01)** | **-0.06* (-0.11, -0.01)** | **-0.06* (-0.11, -0.01)** | -0.05 (-0.10, 0.00) |
| Schedule Tribe | -0.01 (-0.09, 0.06) | -0.05 (-0.12, 0.03) | **-0.09* (-0.17, -0.00)** | -0.04 (-0.11, 0.03) | -0.01 (-0.09, 0.07) | 0.00 (-0.06, 0.07) |
| **Economic status** |  |  |  |  |  |  |
| Higher | ref | ref | ref | ref | ref | ref |
| Lower | **-0.05* (-0.09, -0.01)** | **-0.08*** (-0.12, -0.04)** | **-0.08*** (-0.12, -0.03)** | **-0.09*** (-0.13, -0.04)** | **-0.06** (-0.10, -0.02)** | **-0.06** (-0.10, -0.03)** |
| **Health insurance** |  |  |  |  |  |  |
| No | ref | ref | ref | ref | ref | ref |
| Yes | 0.03 (-0.03, 0.09) | 0.03 (-0.03, 0.08) | 0.03 (-0.03, 0.10) | 0.05 (-0.02, 0.12) | 0.04 (-0.03, 0.10) | 0.05 (-0.00, 0.11) |
| **States/Territories** | **Included** | **Included** | **Included** | **Included** | **Included** | **Included** |
| **Constant** | **3.31*** (3.00, 3.62)** | **3.72*** (3.43, 4.00)** | **3.55*** (3.21, 3.89)** | **3.56*** (3.26, 3.87)** | **3.74*** (3.43, 4.05)** | **3.66*** (3.40, 3.92)** |

**Note:** **Significant at p<0.05, **significant at p<0.01, ***significant at p<0.001.*  *Adjusted estimates are based on multivariable regression models including all covariates listed in the table and states/territories (coefficients not shown).*

**Table S3: HSR domains and functional limitations for INPATIENT care**

| **Covariates** | **Inpatient (N=2,358)** | | | | | |
| --- | --- | --- | --- | --- | --- | --- |
|  | **Prompt attention** | **Dignity** | **Communication** | **Confidentiality** | **Choice of providers** | **Quality of amenities** |
| **ADL/IADL** |  |  |  |  |  |  |
| No | ref | ref | ref | ref | ref | ref |
| At least one | **-0.16** (-0.27, -0.05)** | **-0.12* (-0.22, -0.02)** | -0.06 (-0.16, 0.05) | -0.07 (-0.18, 0.04) | **-0.11* (-0.22, -0.01)** | -0.09 (-0.20, 0.02) |
| **Presence of chronic diseases** |  |  |  |  |  |  |
| None | ref | ref | ref | ref | ref | ref |
| Single | 0.04 (-0.09, 0.18) | 0.11 (-0.03, 0.25) | 0.13 (-0.01, 0.27) | **0.17* (0.03, 0.32)** | **0.16* (0.01, 0.30)** | **0.16* (0.02, 0.31)** |
| Multiple | 0.07 (-0.06, 0.19) | 0.10 (-0.04, 0.24) | **0.17** (0.04, 0.29)** | **0.20** (0.06, 0.34)** | **0.15* (0.02, 0.29)** | 0.10 (-0.03, 0.23) |
| **Residence** |  |  |  |  |  |  |
| Rural | ref | ref | ref | ref | ref | ref |
| Urban | 0.02 (-0.10, 0.14) | 0.05 (-0.07, 0.17) | 0.00 (-0.13, 0.13) | 0.10 (-0.05, 0.25) | 0.04 (-0.10, 0.17) | 0.04 (-0.09, 0.17) |
| **Gender** |  |  |  |  |  |  |
| Female | ref | ref | ref | ref | ref | ref |
| Male | **-0.17** (-0.28, -0.05)** | -0.05 (-0.17, 0.07) | -0.05 (-0.16, 0.06) | -0.05 (-0.18, 0.07) | -0.04 (-0.15, 0.08) | **-0.17** (-0.29, -0.05)** |
| **Age** | -0.00 (-0.01, 0.01) | -0.01 (-0.01, 0.00) | -0.00 (-0.01, 0.00) | -0.00 (-0.01, 0.00) | -0.00 (-0.01, 0.00) | -0.00 (-0.01, 0.01) |
| **Education level** |  |  |  |  |  |  |
| No schooling | ref | ref | ref | ref | ref | ref |
| Less than 5 year complete | -0.08 (-0.27, 0.10) | -0.04 (-0.22, 0.14) | 0.04 (-0.16, 0.24) | 0.08 (-0.13, 0.28) | 0.05 (-0.15, 0.24) | 0.04 (-0.15, 0.22) |
| 5-9 years complete | 0.09 (-0.05, 0.22) | -0.03 (-0.16, 0.11) | -0.01 (-0.15, 0.12) | -0.03 (-0.19, 0.12) | -0.00 (-0.14, 0.13) | -0.04 (-0.19, 0.10) |
| 10 or more years complete | 0.15 (-0.01, 0.32) | **0.24** (0.09, 0.40)** | **0.24** (0.09, 0.39)** | **0.18* (0.01, 0.36)** | **0.23** (0.08, 0.39)** | **0.24** (0.08, 0.40)** |
| **Marriage** |  |  |  |  |  |  |
| Currently not married | ref | ref | ref | ref | ref | ref |
| Currently married | 0.02 (-0.11, 0.15) | 0.02 (-0.11, 0.15) | -0.01 (-0.13, 0.12) | -0.06 (-0.19, 0.07) | -0.06 (-0.19, 0.07) | 0.08 (-0.05, 0.21) |
| **Religion** |  |  |  |  |  |  |
| Hindu | ref | ref | ref | ref | ref | ref |
| Muslim | **0.18* (0.01, 0.34)** | 0.07 (-0.10, 0.24) | 0.08 (-0.11, 0.27) | 0.04 (-0.17, 0.24) | 0.14 (-0.03, 0.32) | 0.12 (-0.08, 0.31) |
| Others | **-0.25* (-0.48, -0.01)** | **-0.30* (-0.60, -0.01)** | **-0.37** (-0.62, -0.12)** | -0.22 (-0.48, 0.03) | **-0.27* (-0.51, -0.03)** | -0.26 (-0.54, 0.02) |
| **Caste** |  |  |  |  |  |  |
| Upper Castes | ref | ref | ref | ref | ref | ref |
| Schedule Caste | -0.02 (-0.16, 0.11) | -0.07 (-0.22, 0.09) | **-0.17** (-0.30, -0.04)** | **-0.19* (-0.34, -0.04)** | **-0.16* (-0.30, -0.02)** | **-0.19* (-0.34, -0.04)** |
| Schedule Tribe | -0.01 (-0.26, 0.23) | -0.05 (-0.31, 0.21) | **-0.19* (-0.37, -0.01)** | **-0.32* (-0.59, -0.05)** | -0.08 (-0.36, 0.20) | -0.09 (-0.33, 0.15) |
| **Economic status** |  |  |  |  |  |  |
| Higher | ref | ref | ref | ref | ref | ref |
| Lower | -0.1 (-0.21, 0.01) | **-0.15* (-0.26, -0.03)** | -0.04 (-0.15, 0.07) | **-0.13* (-0.25, -0.01)** | **-0.16** (-0.28, -0.05)** | **-0.15* (-0.27, -0.04)** |
| **Health insurance** |  |  |  |  |  |  |
| No | ref | ref | ref | ref | ref | ref |
| Yes | 0.08 (-0.04, 0.19) | -0.06 (-0.19, 0.07) | 0.03 (-0.09, 0.14) | 0.00 (-0.12, 0.13) | -0.07 (-0.20, 0.05) | 0.06 (-0.07, 0.19) |
| **States/Territories** | **Included** | **Included** | **Included** | **Included** | **Included** | **Included** |
| **Constant** | **3.59*** (2.87, 4.31)** | **3.84*** (3.14, 4.53)** | **3.81*** (3.02, 4.60)** | **3.63*** (2.87, 4.39)** | **4.01*** (3.31, 4.71)** | **3.78*** (3.07, 4.50)** |

**Note:** **Significant at p<0.05, **significant at p<0.01, ***significant at p<0.001.*  *Adjusted estimates are based on multivariable regression models including all covariates listed in the table and states/territories (coefficients not shown).*

**Table S4: Association between ADL/IADL difficulties and overall HSR for Public and Private healthcare facilities**

| **Covariates** | **Public Outpatient (N=5,040)** | **Private Outpatient (N=9,536)** | **Public Inpatient (N=999)** | **Private Inpatient (N=1,243)** |
| --- | --- | --- | --- | --- |
|  | **Adjusted Coefficient (95% CI)**  **(Model 4a)** | **Adjusted Coefficient (95% CI)**  **(Model 4b)** | **Adjusted Coefficient (95% CI)**  **(Model 4c)** | **Adjusted Coefficient (95% CI)**  **(Model 4d)** |
| **ADL/IADL** |  |  |  |  |
| No | ref | ref | ref | ref |
| At least one | **-0.70*** (-1.08, -0.32)** | **-0.60*** (-0.89, -0.31)** | **-1.09* (-1.99, -0.19)** | -0.45 (-1.07, 0.17) |
| **Presence of chronic diseases** |  |  |  |  |
| None | ref | ref | ref | ref |
| Single | 0.06 (-0.33, 0.45) | 0.00 (-0.29, 0.29) | 1.02 (-0.00, 2.05) | 0.31 (-0.55, 1.18) |
| Multiple | **-0.62* (-1.14, -0.11)** | -0.24 (-0.58, 0.10) | 0.86 (-0.20, 1.91) | 0.50 (-0.31, 1.30) |
| **Residence** |  |  |  |  |
| Rural | ref | ref | ref | ref |
| Urban | 0.11 (-0.33, 0.54) | 0.27 (-0.05, 0.59) | -0.28 (-1.28, 0.72) | 0.50 (-0.30, 1.30) |
| **Gender** |  |  |  |  |
| Female | ref | ref | ref | ref |
| Male | -0.37 (-0.86, 0.11) | **-0.33* (-0.65, -0.01)** | -0.04 (-0.95, 0.88) | **-0.76* (-1.47, -0.05)** |
| **Age** | 0.02 (-0.01, 0.05) | 0.02 (-0.01, 0.04) | -0.03 (-0.09, 0.03) | 0.01 (-0.04, 0.06) |
| **Education level** |  |  |  |  |
| No schooling | ref | ref | ref | ref |
| Less than 5 year complete | 0.47 (-0.05, 0.99) | 0.20 (-0.29, 0.70) | -0.58 (-1.90, 0.74) | 0.22 (-1.06, 1.49) |
| 5-9 years complete | 0.39 (-0.13, 0.91) | **0.66** (0.24, 1.09)** | -0.03 (-1.18, 1.13) | 0.04 (-0.85, 0.93) |
| 10 or more years complete | **0.98** (0.29, 1.67)** | **1.20*** (0.69, 1.72)** | 1.20 (-0.13, 2.52) | **1.12* (0.20, 2.04)** |
| **Marriage** |  |  |  |  |
| Currently not married | ref | ref | ref | ref |
| Currently married | 0.06 (-0.39, 0.51) | 0.22 (-0.12, 0.55) | -0.44 (-1.44, 0.56) | 0.23 (-0.52, 0.98) |
| **Religion** |  |  |  |  |
| Hindu | ref | ref | ref | ref |
| Muslim | 0.33 (-0.37, 1.03) | 0.01 (-0.37, 0.39) | **1.93* (0.46, 3.39)** | -0.17 (-1.33, 0.99) |
| Others | **-1.18** (-1.87, -0.49)** | -0.49 (-1.04, 0.06) | **-3.58** (-6.17, -0.99)** | -1.10 (-2.64, 0.43) |
| **Caste** |  |  |  |  |
| Upper Castes | ref | ref | ref | ref |
| Schedule Caste | 0.03 (-0.45, 0.51) | -0.20 (-0.55, 0.14) | -0.32 (-1.31, 0.68) | -0.35 (-1.25, 0.55) |
| Schedule Tribe | **0.81* (0.04, 1.58)** | **-0.67** (-1.10, -0.23)** | 0.23 (-1.34, 1.79) | -1.27 (-2.87, 0.33) |
| **Economic status** |  |  |  |  |
| Higher | ref | ref | ref | ref |
| Lower | **-0.90*** (-1.28, -0.52)** | -0.09 (-0.35, 0.16) | **-1.16** (-1.99, -0.32)** | -0.04 (-0.72, 0.63) |
| **Health insurance** |  |  |  |  |
| No | ref | ref | ref | ref |
| Yes | 0.22 (-0.19, 0.62) | 0.32 (-0.14, 0.78) | -0.73 (-1.65, 0.20) | 0.43 (-0.31, 1.17) |
| **States/Territories** | **Included** | **Included** | **Included** | **Included** |
| **Constant** | **20.02*** (17.48, 22.55)** | **23.58*** (21.44, 25.72)** | **22.34*** (17.05, 27.62)** | **26.05*** (21.94, 30.16)** |

***Note:*** **Significant at p<0.05, **significant at p<0.01, ***significant at p<0.001.*  *Adjusted estimates are based on multivariable regression models including all covariates listed in the table and states/territories (coefficients not shown).*
